# South West Urological Association

**Published:** 1984-01

**Authors:** 


### South West Urological Association

Meeting held at Southmead Hospital, Bristol, 30th September 1983

### RENAL TRANSPLANTATION IN CHILDREN

B. D. Pentlow, and T. L. Chambers, Southmead Hospital, Bristol.

Recent advances have proved advantageous in paediatrics and renal transplantation.

Continuous Ambulatory Peritoneal Dialysis (CAPD) facilitates dialysis of low weight children including babies. The beneficial effects of pre-transplant blood transfusions are appreciated, likewise donor specific blood transfusions in the case of living donors.

DR typing has a stronger correlation with graft success than conventional tissue typing. The use of lower doses of steroids reduces the side effects and growth retardation in children. The new immunosuppression agent Cyclosporin A may prove beneficial.

Examples of successful paediatric kidney transplants at Southmead illustrating these features were presented ranging upwards in age from 6 years. The causes of renal failure varied from congenital 'reflux nephropathy' to Henoch–Schönlein's purpura.

Two failures were described. A girl of 12 received a transplant that was initially very successful, only to suffer a recurrence of her original disease (mesangiocapillary glomerulo-nephritis Type II) in the graft. A 7-kg baby transplanted with some reservations lost its graft from a ureteric leak. The transplanted kidney of a 14-year-old boy, however, was saved by the use of a Boari flap after it had sloughed its entire ureter.

Two girls aged 7 and 12 who had 'occult neurogenic' bladders managed by self-catheterisation received successful transplants into their bladders with no undue problems of infection.

A girl whose ureters were diverted at the age of 10 months for 'mega ureter, mega cystitis' received a transplant draining into her bladder more than 16 years later. There were no problems of micturition.

A Jehovah's witness who had exhausted her dialysis sites received a successful graft from a donor of 72 without blood transfusion.

### ENDOSCOPIC BLADDER NECK SUSPENSION FOR STRESS INCONTINENCE

Paul H. Abrams, Southmead Hospital, Bristol

The basis of surgical treatment for genuine stress incontinence is the repositioning of the bladder neck within the abdominal cavity. A variety of procedures have been devised in order to accomplish this aim. In general, operations via the suprapubic approach have a more successful outcome when assessed by objective methods, including Urodynamics.

Endoscopic bladder neck suspension (Stamey technique) uses an anterior vaginal incision and two small suprapubic incisions to position a nylon sling on either side of the bladder neck. From the vaginal approach small pouches are developed on either side of the bladder neck. A specially designed needle is passed via the suprapubic incision behind the pubis to emerge into the vagina via the anterior wall incision. The needle is used to pull a nylon suture through to the anterior abdominal wall. A 1-cm length of silastic tube is threaded on to the nylon and pulled through by the needle passed behind the pubis for the second time. Thus the two ends of the nylon loop emerge from the suprapubic incisions whilst the silastic buttress rests in the paraurethral tissues at the bladder neck. The procedure is repeated on the other side of the bladder neck. After each passage of the needle the bladder is endoscoped to ensure the needle has remained outside the urinary tract. The ends of the loop are tied down to the anterior rectus sheath with sufficient tension to prevent stress incontinence.

To date 15 operations have been performed on women who had undergone previous failed gynaecological surgery. Eleven have been successful and 4 have failed. Morbidity has been low. This technique appears particularly helpful in the old and infirm.

### URINARY INCONTINENCE IN WOMEN TREATED BY PERIURETHRAL TEFLON INJECTION

R. C. L. Feneley, Southmead Hospital, Bristol

The use of Teflon in the treatment of urinary incontinence was first described by Berg (1973) and then by Politano (1974). The injection of the viscous paste into the urethra and periurethral tissues aims to increase the urethral resistance in patients whose urinary incontinence is related to a reduced urethral pressure. This method has been used particularly in those women whose initial operative treatment had failed to control the urinary leakage. Detailed assessment, including urodynamic studies, is essential to select those patients suitable for this approach.

The injection of the Polytef paste (Ethicon) is given at the time of cystoscopy under general anaesthesia. Following examination of the bladder, a 0 degree telescope is used to visualise the bladder neck and proximal urethra. A 1.7 mm intravenous medicut Argyle needle with cannula is introduced between the external urethral meatus and vagina and advanced between these structures to the level of the bladder neck. The needle is removed and a 5 ml syringe filled with the paste is attached to the cannula. Injection requires strong pressure and the aim is to create a bulge on the posterior aspect of the bladder neck. Care is needed to avoid penetration or undue pallor of the mucosa during the injection. Further injections are then given at the lateral aspects of the bladder neck on either side, thus producing the final appearance similar to that of middle and lateral lobe enlargement of the prostate gland. A total of 15–20 ml of Polytef is used.

The results of an initial series of patients were outlined by Lim et al. in 1983. Twenty-eight female patients were treated by this method, of whom 26 had previously undergone operative treatment for urinary incontinence. Six patients were cured of their incontinence following the injection and 9 were temporarily improved. Thirteen patients experienced no change. If patients with detrusor instability had been excluded from this series, the results would have been improved.

The use of Teflon paste in the treatment of women with urinary incontinence is a simple procedure. The duration of stay in hospital is short and usually less than 4 days. If the initial treatment is not successful, a repeat injection can be given. The Polytef paste causes a foreign body giant cell reaction at the site of the injection. The best results were achieved in those patients who showed a high cystometric capacity and a low maximum urethral pressure on urodynamic investigation.

### SURGERY FOR POST-COITAL CYSTITIS: THE O'DONNELL OPERATION

**A. J. Ball, Bristol**

Many urologists are regularly faced with the problem of the female with recurrent frequency and dysuria in whom 'conservative' treatment, including numerous courses of antibiotics, has failed. This procedure has been used to treat a small number of patients whose urinary symptomatology is unquestionably post-coital. The sound theoretical basis for this approach is precisely defined on anatomical grounds. A relative 'hypospadias' of the external urethral meatus results from persistent hymenal remnants tethering the urethra into an intravaginal position. A ventral hood is frequently present over the meatus, and the net effect of these abnormalities is to render the patient likely to develop the condition known as 'traumatic transvaginal cysto-urethritis' as a result of sexual activity. Such patients have urethral pain during intercourse (urethral dyspareunia), unexplained on grounds of bacterial contamination alone.

The minor procedure to release the urethra anteriorly, lessening its traumatisation during intercourse, is described. It is essential that no symptoms had occurred prior to the onset of sexual activity, abstinence from which characteristically produces relief, and that the physical signs outlined can be demonstrated. Results thus far in Bristol are very encouraging.

### EARLY PROSTATIC CANCER DIAGNOSIS AND TREATMENT

**J. C. Gingell, Southmead Hospital, Bristol**

The incidence of unsuspected or incidental carcinoma of the prostate found at transurethral resection for clinically benign obstructive disease is high and increases steadily with the age of the patient. Systematic sectioning of the prostate in men dying of another disease has shown that at least 30% of men over the age of 50 years have carcinoma of the prostate. There is a very large discrepancy, however, between those who have histological evidence of the disease and those who actually die from it. Is this due to latency or biological inactivity of the tumour or to a growth rate so slow that the condition usually does not present as a clinical problem? If one considers the often advanced age of the patient in whom incidental disease is diagnosed, then it can be appreciate that they are much more likely to die from another cause. The progress of 26 patients with incidental carcinoma of the prostate, age 61–89 years (mean 74 years) followed for 2–9 years (mean 3.9 years), in whom no initial specific anti-cancer treatment was undertaken was reported. There were 4 deaths from unrelated causes. There were 2 patients, 3 years after initial diagnosis, who because of local progression of the carcinoma causing symptoms required a further TUR with bilateral orchidectomy in one case and Stilboestrol in the other. Both are alive and well at 2 years and 6 years respectively. One patient developed metastases 3 years after initial diagnosis for which he received Stilboestrol but died 9 months later. The remaining patients are alive and well with no urinary symptoms or evidence of metastases. Deferred treatment for early stage well-differentiated incidental carcinoma of the prostate is acceptable in the older patient provided close follow up is maintained.

### EXTERNAL BEAM RADIOTHERAPY FOR CARCINOMA OF THE PROSTATE GLAND IN THE BRISTOL AND BATH CLINICAL AREA

**A. V. Kaisary, C. C. Gaffney, H. Eckert, P. J. B. Smith and J. C. Gingell, Royal Infirmary, Bristol**

A retrospective survey has revealed that 116 patients have received radical radiotherapy for carcinoma of the prostate at the Bristol Radiotherapy Centre between January 1972 and December 1979. For the purpose of this paper, an attempt has been made to identify those patients with locally confined disease (SAP normal, Bone Scan negative) who received treatment during this time including the period until 1981. Forty-four patients meeting these criteria were reviewed. The age range at presentation was 48–80 years (mean 66.7 years). The presenting symptoms in 39 patients were suggestive of outflow obstruction (88.7%), haematuria in 2 patients (4.5%) and one patient with impotence (2.3%). Two patients were symptom-free (4.5%). A review of the histological grade and clinical stage showed a tendency towards poor differentiation in the more advanced clinical stages (T3–T4). The usual side effect of radiotherapy was that of diarrhoea and blood/mucous per rectum (65.9%). Review of treated patients showed persistence of mild diarrhoea with occasional bleeding per rectum in 11.3%.

The overall results of treatment demonstrated successful control of the disease in 25 patients who are alive and well (range 0.5–5.7 years, mean 3.1 years). Sixteen patients have died, however, due to prostatic carcinoma (range of 0.7–3.5 years, mean 1.3 years). Three patients were lost to follow-up. Twelve patients have been regularly reviewed after radiotherapy using the Franzen prostatic needle aspiration technique. The results of cytological examination correlated well with the clinical status of the disease and is proving to be useful in predicting the response to treatment.

### CRYOSURGERY OF THE PROSTATE

**R. G. Hughes, A. P. Wetherall, M. J. Cooper and I. Sutherland-Jones, Plymouth General Hospital, Plymouth**

Between 1969 and 1981, 245 patients underwent cryoprostatectory under the care of one surgeon (I. S.-J.). Their median age was 74 (range 46–95) and 148 patients presented in acute retention. Only 18 glands were thought clinically to be malignant. Using the American Society of Anesthetists grading of preoperative fitness, only 81 patients (33%) were assessed as grade 1, and many patients were specifically referred for this procedure because of severe concurrent illness. Most cryosurgery was performed under general anaesthesia, and the cryoprobe reached −180°. The duration of freezing was varied according to the size of the gland. A catheter was inserted after freezing and remained in situ for 3 weeks. When the catheter was removed 75% of patients were able to void satisfactorily. Thirty-five patients went into retention, but after transurethral resection of prostatic slough or a further period of catheterisation passed water satisfactorily. Incontinence and dribbling were noted after catheter removal but resolved spontaneously. Finally, 91.5% of patients treated were voiding satisfactorily. The catheter was not removed from 6 patients because of severe and advancing concurrent disease. At follow-up at least 6 months postoperatively, 4 patients were noted to have developed urethral strictures and 3 had a permanent indwelling catheter. Sixteen patients had died in this period for reasons not associated with the operation.

The overall mortality for the operation was 4.8% and 2.45% of patients experienced complications. However, when those patients whose death was not attributable to the operation were discounted, the mortality fell to 1.63%. Cryoprostatectomy is a satisfactory and safe method of dealing with prostatic hypertrophy in an elderly and infirm age group.

### INTERSTITIAL IODINE 125 IN THE TREATMENT OF LOCALISED PROSTATIC CANCER

**Patrick Smith, Royal Infirmary, Bristol**

Prostatic cancer is now the second commonest tumour in men in the United Kingdom. The incidence in the South West is the highest in the land. Treatments for this disease are many and varied and must be considered in each case in relation to the individual patients. However, there are a group of men in whom the disease is detected early and without any obvious metastases. These patients should have some form of attempted curative therapy. In the past this has consisted of external beam radiotherapy. Though the results of this in terms of cure and, more importantly, local tumour control are excellent, complications are significant. For this reason the Department of Urology at the Bristol Royal Infirmary, in conjuction with the Radiotherapy Centre, has developed a technique of interstitial iodine 125 therapy. This treatment produces a high dose of local radiotherapy over a 6-month period. The radioactivity is emitted by a small tantalum seed encasing a radioactive iodine source. The seeds are inserted through cannula using an open operative technique. This involves a retropubic dissection of the prostate together with mobilisation of the pelvic floor to enable the whole gland to be visualised. Technique does cause some bleeding but this has not proved a problem to date. In all 10 patients have been treated in this way. All are alive and well, with no evidence of metastatic disease. Local control has been excellent. Patients submitted for this treatment should be biologically fit, should not have metastatic disease and should be free of any lymph node involvement. To this effect pre-treatment bone scan, lymphangiogram and CT scans are essential. Newer techniques will involve percutaneous insertion of these seeds under ultrasound control or possible, eventually, transurethral insertion. The use of Iodine 125 in the management of prostatic cancer has opened up a new and attractive area of treatment of early stage prostatic malignancy.

### THE ROLE OF VIDEO-CYSTOURETHROGRAPHY

**J. A. Massey, Ham Green Hospital, Bristol**

The procedure was first described in the 1960s. Modern innovations include analogue-video converters, micro-computers and ultrasonography. These studies give the best overall assessment of urodynamic function at all stages of the filling and voiding cycle. Careful selection, however, should be employed in view of unnecessary irradiation, cost considerations and complexity. Further, the video element contributes nothing in cases of simple obstruction, urgency or stress incontinence. The indications we use, examples of which were shown on videotape, are

(i) atypical outflow tract obstruction (covert neuropathic bladders, bladder neck dysfunction and distal urethral obstruction),
(ii) children,
(iii) complicated incontinence (e.g. after previous surgery),
(iv) neurological problems, and
(v) when a simple urodynamic study does not give a clearcut answer.

The advantages are the ability to see bladder and urethral morphology and the presence of reflux. Only these studies show bladder neck dyssynergia or incompetence, and dyssynergic distal sphincter with normal pelvic floor synchronisation or accurately localise urethral obstruction.

### THE USE OF URODYNAMICS IN THE INVESTIGATION OF VOIDING DISORDERS IN CHILDREN

**J. D. Frank and P. Abrams, Southmead Hospital, Bristol**

Considerable controversy exists as to the value of urodynamic investigations in children. It is our experience that there are children in whom this investigation is not only invaluable, but necessary.

The aims of the investigation are
1. The assessment of sphincter continence.
2. An estimate of bladder compliance.
3. An estimate of detrusor activity.
4. The assessment/exclusion of outflow obstruction.

Techniques are standardised so that the children are admitted 24 hours before the investigation and a suprapubic catheter inserted at cystoscopy. Sedation is not given and repeated investigations are performed until comparable results are obtained.

Urodynamics have been found to be valuable in the following groups.

1. Spina bifida to assess;
   (a) bladder function prior to intermittent self-catheterisation,
   (b) bladder function prior to urinary undiversion,
   (c) bladder function prior to the insertion of an artificial sphincter.
2. Children with voiding dysfunction.
3. Bladder assessment prior to renal transplantation.
4. The assessment of bladder function in patients with deteriorating upper renal tracts.

Four patients were presented who illustrated the value of urodynamics and investigations in these diagnostic groups.

### SIMPLE OUTPATIENT URINE FLOWMETRY

**Dr. M. Lucarotti, Bristol**

'Prostatism' is a syndrome comprising frequency of micturition, nocturia, urgency, incontinence, slow stream, hesitancy and post-micturition dribble. More and more patients are being referred with these symptoms and a simple screening test is therefore required.

The urine flow meter provides an objective measurement of voiding dysfunction.

A study of 60 male patients has been made who were referred with one or more of the symptoms of prostatism. Measurement of their peak urine flow rates were used to attempt definition of those with possible outflow obstruction.

Results showed that 82% of the patients were aged over 55 years and 55% of these were clinically said to have an enlarged prostate. Only 19 (32%) had a flow rate of 12 ml/s or less (2 standard deviations below the norm on the Siroky nomogram). Of these, 13 patients proceeded to prostatectomy, 2 had urethral strictures, and 4 improved spontaneously or with Phenoxybenzamine.

The estimation of urine flow rates is an accepted method of evaluating bladder outflow obstruction. This presentation outlines the value of a urine flow clinic and describes the regime used.

